# Myricetin, the Main Flavonoid in *Syzygium cumini* Leaf, Is a Novel Inhibitor of Platelet Thiol Isomerases PDI and ERp5

**DOI:** 10.3389/fphar.2019.01678

**Published:** 2020-01-31

**Authors:** Renato Simões Gaspar, Samira Abdalla da Silva, Jennifer Stapleton, João Lucas de Lima Fontelles, Hiran Reis Sousa, Vinicyus Teles Chagas, Shuruq Alsufyani, Andrés Trostchansky, Jonathan M. Gibbins, Antonio Marcus de Andrade Paes

**Affiliations:** ^1^ Institute for Cardiovascular and Metabolic Research, School of Biological Sciences, University of Reading, Reading, United Kingdom; ^2^ Laboratory of Experimental Physiology, Department of Physiological Sciences, Federal University of Maranhão, São Luís, Brazil; ^3^ Departamento de Bioquímica and Centro de Investigaciones Biomédicas, Facultad de Medicina, Universidad de la República, Montevideo, Uruguay

**Keywords:** *Syzygium cumini*, antithrombotic agents, platelet, oxidation-reduction, platelet aggregation inhibitors

## Abstract

**Background:**

Flavonoids have been characterized as a prominent class of compounds to treat thrombotic diseases through the inhibition of thiol isomerases. *Syzygium cumini* is a flavonoid-rich medicinal plant that contains myricetin and gallic acid. Little is known about the potential antiplatelet properties of *S. cumini* and its constituent flavonoids.

**Objective:**

To evaluate the antiplatelet effects and mechanism of action of a polyphenol-rich extract (PESc) from *S. cumini* leaf and its most prevalent polyphenols, myricetin and gallic acid.

**Methods:**

PESc, myricetin, and gallic acid were incubated with platelet-rich plasma and washed platelets to assess platelet aggregation and activation. *In vitro* platelet adhesion and thrombus formation as well as *in vivo* bleeding time were performed. Finally, myricetin was incubated with recombinant thiol isomerases to assess its potential to bind and inhibit these, while molecular docking studies predicted possible binding sites.

**Results:**

PESc decreased platelet activation and aggregation induced by different agonists. Myricetin exerted potent antiplatelet effects, whereas gallic acid did not. Myricetin reduced the ability of platelets to spread on collagen, form thrombi *in vitro* without affecting hemostasis *in vivo*. Fluorescence quenching studies suggested myricetin binds to different thiol isomerases with similar affinity, despite inhibiting only protein disulfide isomerase (PDI) and ERp5 reductase activities. Finally, molecular docking studies suggested myricetin formed non-covalent bonds with PDI and ERp5.

**Conclusions:**

PESc and its most abundant flavonoid myricetin strongly inhibit platelet function. Additionally, myricetin is a novel inhibitor of ERp5 and PDI, unveiling a new therapeutic perspective for the treatment of thrombotic disorders.

## Introduction

Cardiovascular diseases are the leading cause of death worldwide, a scenario where thrombosis and its associated outcomes account for one in four deaths (Wendelboe and Raskob, 2016). Platelets play a key role in arterial thrombosis, due to platelet aggregation triggered by multiple agonists, such as adenosine diphosphate (ADP), thrombin, and collagen. These signaling pathways will inevitably culminate in the activation of the platelet surface integrin αIIbβ_3_ (Banno and Ginsberg, 2008; Ghoshal and Bhattacharyya, 2014), which becomes activated after the isomerization of critical disulfide bonds on its extracellular β domain. This process is thought to be mediated by protein disulfide isomerase (PDIA1, herein referred to as PDI) and sibling proteins (Essex, 2008). Therefore, PDI has been proposed as a new target to treat and prevent thrombotic diseases (Jasuja et al., 2012).

PDI is the leading member of its family, a set of thioredoxin-like thiol isomerases originally described in the endoplasmic reticulum (ER), but later found in virtually all cell compartments, including the platelet surface (Essex et al., 1995). In platelets, PDI has been shown to regulate integrins αIIbβ_3_ and α_2_β_1_, the latter being a collagen receptor important for platelet adhesion (Lahav et al., 2003; Essex, 2008). Besides PDI, at least three other members—ERp5 (PDIA6), ERp57 (PDIA3), and ERp72 (PDIA4)—have been demonstrated to support thrombosis (Essex and Wu, 2018). Particularly, ERp5 has been implicated in integrin αIIbβ_3_ activation and shown to become physically associated with integrin β_3_ upon platelet activation (Jordan et al., 2005). Therefore, there has been a surge of novel PDI inhibitors being described, both synthetic (Sousa et al., 2017) and natural, such as the flavonoid quercetin and its derivatives (Lin et al., 2015). Accordingly, flavonoids and related compounds have been described as potent antiplatelet compounds, acting through diverse mechanisms (Jasuja et al., 2012; Giamogante et al., 2018).

*Syzygium cumini* (L.) Skeels (Myrtaceae) is a worldwide cultivated medicinal plant, popularly known as jamun, black plum, jambolan, or jambolão (Ayyanar and Subash-Babu, 2012). *S. cumini* has been proposed as a prominent source of bioactive compounds against cardiometabolic disorders (Chagas et al., 2015), in accordance with its usage in the Unani medicine to “enrich blood” (Ayyanar and Subash-Babu, 2012). Indeed, *S. cumini* has been shown to inhibit the hyperactivation of platelets from diabetic patients (De Bona et al., 2010; Raffaelli et al., 2015). Recently, we characterized a polyphenol-rich extract from *S. cumini* (PESc) leaf, which consisted of gallic acid, quercetin, myricetin, and its derivatives myricetin-3-α-arabinopyranoside and myricetin deoxyhexoside (Chagas et al., 2018). Of the flavonoids identified, myricetin was the most abundant, constituting roughly 20% of PESc weight (Chagas et al., 2018). Interestingly, this extract has been shown to reduce oxidative stress and prevent the development of diabetes induced by alloxan treatment (Chagas et al., 2018). Despite this, there is scarce literature on the antiplatelet properties of *S. cumini* and its most abundant polyphenols, myricetin and gallic acid.

Therefore, in the present study, we hypothesized that PESc presents potential antiplatelet properties and that myricetin and gallic acid, as the most prevalent compounds, would be its bioactive phytochemicals. Moreover, given the structural similarity between myricetin and quercetin, we also tested for a possible inhibition of thiol isomerases. Data herein presented endorse our hypothesis through the demonstration of PESc inhibitory effects on both platelet activation and aggregation. Assessment of gallic acid and myricetin bioactivity showed that only myricetin exerted physiologically relevant antiplatelet properties. Myricetin was then shown to be a novel inhibitor of thiol isomerases PDI and ERp5, unveiling a new therapeutic perspective for the treatment and prevention of thrombotic diseases.

## Materials and Methods

### Reagents

Myricetin, gallic acid, ADP, thrombin, phorbol-12-myristate-13-acetate (PMA), Thrombin Receptor Activator Peptide 6 (TRAP-6), human fibrinogen, and 1,4-Dithiothreitol (DTT) and 3,3′-Dihexyloxacarbocyanine iodide (DIOC_6_) were purchased from Sigma-aldrich (Dorset, UK). PAPA-NONOate was purchased from Tocris (Abingdon, UK). PE/Cy5 anti human CD62P and PAC-1 FITC antibodies were purchased from BD Biosciences (Wokingham, UK). FITC-conjugated fibrinogen was purchased from Agilent (Stockport, UK). Collagen was purchased from Nycomed (Munich, Germany) whereas Collagen-Related Peptide (CRP) was obtained from Prof Richard Farndale (University of Cambridge, Cambridge, UK). Anti-phospho-vasodilator-stimulated phospho-protein (VASP) (Ser239) was purchased from Cell Signalling (Hitchin, UK), anti-glyceraldehyde 3-phosphate dehydrogenase (GAPDH) from Proteintech (Manchester, UK), and Alexa-488 conjugated phalloidin secondary antibody was bought from Life Technologies (Paisley, UK)

### Botanical Material

*S. cumini* leaves were collected from different trees at the campus (2°33´11.7´´S 44°18´22.7´´W) of the Federal University of Maranhão (UFMA) in São Luís, Maranhão, Brazil. Samples were identified and catalogued at the Herbarium MAR of the Department of Biology of the same institution, where a voucher specimen was deposited under n° 4573.

### Extract Preparation

The extract was prepared according to Sharma et al. (2008), with modifications. Fresh leaves were dried at 38°C in an air-flow oven, pulverized into powdered dry leaves (150 g), and macerated in 70% ethanol (1:6 w/v) under constant stirring for 3 days at 25°C. The supernatant was concentrated in a rotary evaporator to obtain the crude hydroalcoholic extract (HE). HE was partitioned with chloroform (1:1 v/v, 3x) and the organic phase was washed with ethyl acetate (1:1 v/v, 3x). The ethyl acetate fraction was concentrated under vacuum (38°C) and lyophilized, yielding the polyphenol-rich extract (PESc). For experimental procedures, PESc samples were resuspended in water, at desired concentrations, immediately before use.

### Confirmation of Polyphenolic Composition of PESc

As we have previously characterized the phytochemical composition of PESc (Chagas et al., 2018), confirmatory assessment was performed by both HPLC-UV/Vis detection and LC-MS/MS to validate the lot of PESc used in this study. Methods employed were exactly as previously described (Chagas et al., 2018).

### Platelet-Rich Plasma and Washed Platelets Preparation

Healthy volunteers who did not use antiplatelet drugs and had previously provided informed consent had their blood samples collected in tubes containing 1:5 v/v acid citrate dextrose (ACD: 2.5% sodium citrate, 2% D-glucose, and 1.5% citric acid) or 3.8% (w/v) sodium citrate for platelet aggregation experiments using platelet-rich plasma (PRP). Whole blood was centrifuged at 250 × *g* for 10 min at 22°C to obtain PRP. To obtain washed platelets (WP), PRP was centrifuged twice (1,000 × *g*, 10 min, 20°C) in the presence of 1.25 μg/ml prostacyclin. The final platelet pellet was resuspended in modified Tyrode’s-HEPES buffer, (20 mM N-2-hydroxyethylpiperazine-N′-2-ethanesulfonic acid, 5 mM glucose, 134 mM NaCl, 0.34 mM Na_2_HPO_4_, 2.9 mM KCl, 12 mM NaHCO_3,_ and 1 mM MgCl_2_, pH 7.3) and rested for 30 min at 30°C before experiments. All protocols using human blood samples were approved by the Research Ethics Committees of the Federal University of Maranhão and the University of Reading.

### Platelet Aggregation

PRP and WP aggregation assays were performed in a four-channel aggregometer (Helena Biosciences, Gateshead, England). PRP samples (2–3 × 10^8^ platelets/ml) were incubated for 25 min at 37°C with 10, 100, or 1,000 μg/ml of PESc prior to the addition of ADP (2.5 or 5 µM), thrombin (0.01 or 0.02 U/ml), or PMA (100 nM). For experiments using myricetin and gallic acid, these were incubated in PRP (10, 30, or 100 µM for myricetin and 100, 300, or 1,000 µM for gallic acid) or WP (7.5, 15, or 30 µM for myricetin and 75, 150, or 300 µM for gallic acid) for 10 min at 37°C followed by the addition of agonists collagen (1 µg/ml) or TRAP-6 (10 µM). Aggregation traces from at least three different donors were recorded for 5 min.

### Flow Cytometry

WP (2–3 × 10^8^ platelets/ml) were incubated with PESc at the same concentrations and conditions used for platelet aggregation experiments. Then WP were incubated for 10 min with thrombin (0.02 U/ml). FITC-conjugated PAC-1 antibody was added for 10 min in the dark and fluorescence read using a flow cytometer FACS Calibur (BD Biosciences, Franklin Lakes, USA). For experiments using myricetin and gallic acid, these were incubated with WP for 10 min at 37°C followed by the addition of agonists CRP (1 µg/ml) or TRAP-6 (10 µM). FITC-conjugated fibrinogen and PE/Cy5-conjugated anti-human CD62P were incubated for 30 min, then platelets were read after a 25-fold dilution with Tyrodes-HEPES buffer.

### Platelet Spreading

WP (2 × 10^7^ platelets/ml) were incubated in absence or presence of myricetin (7.5, 15, and 30 µM) for 10 min at 37°C, then 300 µl of the solution was dispensed onto a fibrinogen or collagen (100 µg/ml)-coated coverslip for 45 min at 37°C. Non-adhered platelets were removed and the coverslip washed three times with PBS. Adhered platelets were fixed using 0.2% paraformaldehyde for 10 min. This solution was then removed and coverslips washed three times with PBS before the addition of 0.1% (v/v) Triton-X to permeabilize the cells. After removal of Triton-X and further washes using PBS, platelets were stained with Alexa Fluor 488 or 647-conjugated phalloidin (1:1,000 v/v) for 1 h in the dark at room temperature. Coverslips were mounted onto microscope glass slides and imaged using a 100× oil immersion lens on a Nikon A1-R Confocal microscope.

### Thrombus Formation Under Flow

Whole blood was pre-incubated with DIOC_6_ (5 µM) for 30 min at 30°C, whilst Vena8 bio-chips (Cellix Ltd, Dublin, Ireland) were coated with collagen (100 µg/ml) for 60 min at 37°C. Prior to experiments, blood was pre-treated with myricetin (30 µM) or vehicle control for 10 min at 37°C and Vena8 bio-chips were washed with Tyrode’s-HEPES buffer. Whole blood was then perfused at a shear rate of 45 dyn/cm^2^ and images recorded every 4 s for 10 min using a 20× air lens on a Nikon A1-R Confocal microscope exciting at 488 nm and detecting emission at 500 to 520 nm. Fluorescence intensity was calculated using NIS Elements Software (Nikon, Tokyo, Japan).

### Tail Bleeding Assay

Healthy female Swiss mice (*mus musculus)* with 10–12 weeks of age and 30–35 grams were acquired from the Animal Facility House of the Federal University of Maranhão (UFMA), São Luís–MA. Animals were kept under a 12 h light cycle, controlled temperature (22–24°C) and food and water *ad libitum*. Mice were given myricetin at 25 mg/kg or 50 mg/kg or vehicle control for three consecutive days through oral gavage. One hour after the last dose, animals were anesthetized with ketamine (100 mg/kg) and xylazine (10 mg/kg) and 5 mm of the tail was amputated using sharp scissors. The bleeding tail was then placed in filtered PBS buffer at 37°C and time to cessation of bleeding recorded for up to 20 min, after which mice were terminated. All procedures were performed in alignment with the National Council for the Control of Animal Experimentation (CONCEA, Brazil) and approved by the local Animal Care and Welfare Committee of UFMA, under ruling number 23115.018725/2017-19.

### Generation of Full-Length Recombinant Erp5, Erp57, Erp72, and PDI

The generation of recombinant thiol isomerases was performed as previously described (Holbrook et al., 2018). Briefly, cDNA for ERp5, ERp57, ERp72, and PDI were subcloned into pGEX6P1 expression vector in *Escherichia coli* to generate a glutathione s-transferase (GST)-tagged fusion protein. The fusion protein was then purified by affinity chromatography using glutathione agarose and the GST cleaved using PreScision protease as per manufacturer instructions (GE Healthcare, Amersham, UK). Finally, the proteins were submitted to a gel filtration on Superdex 75 purification resin (GE Healthcare, Amersham, UK) to remove contaminants.

### Protein Quenching Analysis and Biochemical Equations

Myricetin (0.01–10 µM) or vehicle (1:400 v/v DMSO : PBS) were incubated with recombinant ERp5, ERp57, ERp72, or PDI (2 µM) in PBS buffer containing ethylenediaminetetracetic acid (EDTA, 0.2 mM) for 10 min at 25°C in a black 96-well plate. Fluorescence intensity was read using a Flexstation 3 fluorimeter (Molecular Devices, Wokingham, UK), with 280 nm excitation and emission scanned from 300 to 420 nm in 5 nm slits, at a speed of 0.17 s per well. Appropriate blanks in which no protein was added were also acquired to establish that myricetin had no autofluorescence at the specified excitation/emission wavelengths. Data obtained are the means of at least three independent experiments run at least in duplicate.

To calculate the Stern-Volmer quenching constant (*K*_SV_), peak fluorescence intensity at 330 nm was used and constant determined from the following equation:

(1) $$\frac{F_{0}}{F}=1+K_{\text{SV}}\left[L\right]$$

where *F*_0_ is the fluorescence of protein alone, *F* is the fluorescence in the presence of increasing concentrations of myricetin, and *L* is the concentration of myricetin used. *K*_SV_ was then calculated as the slope from the linear regression of *F*_0_/*F* versus [*L*]. Data is shown as log [*L*]. The quencher rate coefficient *K*q was determined from the formula:

(2) $$K\text{q}=\frac{K_{\text{SV}}}{\tau_{0}}$$

where τ_0_ is the average lifetime of the emissive excited state of the protein in the absence of the inhibitor. Previous reports have determined the typical value of τ_0_ to be in the order of 10^-8^ s (Lakowicz and Weber, 1973), which was also adopted for the values herein presented.

The apparent binding constant (*K*_b_) was determined according to the equation of Bi et al. (2005):

(3) $$\log\left(\frac{F_{0}-F}{F}\right)=\;n\text{log}K_{\text{b}}-n\text{log}\left(\frac{1}{\left[L\right]-n\left(F_{0}-F\right)\left[P\right]/F_{0}}\right)$$

In which *F*_0_ is the fluorescence of protein alone, *F* is the fluorescence in the presence of increasing concentrations of myricetin, [*L*] is the ligand concentration, and [*P*] is the protein concentration in M. First, the linear regression of log [(*F*_0_ − *F*)/*F*] versus log [1/([*L*] − *n* (*F*_0_ − *F*)[*P*]/*F*_0_)] was plotted and *n* determined as the slope of the regression, as described by Bi et al. (2005). Then, *n* was substituted back into the equation and *K*b determined for the highest concentration tested. Finally, the dissociation constant (*K*_d_) was calculated as *K*_d_ = 1/*K*_b_.

### Reductase Activity

The reductase activity of the thiol isomerases was determined through the fluorescent probe dieosin glutathione disulfide (Di-E-GssG, excitation: 510 nm, emission: 545 nm). Di-E-GssG was synthesized and used as previously described (Raturi and Mutus, 2007). Myricetin (0.01–10 µM) was incubated for 10 min with recombinant proteins (2 µM) diluted in PBS and EDTA (2 mM) buffer in a 96-wells black plate. Then, DTT (5 µM) and Di-E-GssG (200 nM) were added and fluorescence intensity acquired on a Flexstation 3 fluorimeter (Molecular Devices, Wokingham, UK). Fluorescence intensity was acquired every 30 s for 30 min. Data presented are the means of at least three independent experiments run at least in duplicate.

### Molecular Docking

The predicted poses of interaction between myricetin and different thiol isomerases were assessed using AutoDock 4.2 package, similar to previously described (Wang et al., 2018). The 3D structures of proteins were obtained from the PDB database (PDB ID: 4EL1 for PDIA1 and PDB ID: 4GWR for PDIA6/ERp5). The grid box of analysis was set as a perfect cube of 20 × 20 × 20 points with 1.00 Å spacing centered at the tryptophan residue near the active site of each thiol isomerase and the exhaustiveness of the runs set to 128. The 20 predicted poses with the best binding affinity were generated for each protein and each pose was studied individually to assess if chemically sound using Pymol software (Schrodinger, Cambridge, UK).

### Immunoblotting

WP (4 × 10^8^ platelets/ml), were incubated with myricetin (7.5, 15, and 30 µM) or the nitric oxide donor PAPA-NONOate (100 µM), lysed in reducing Laemmli sample buffer [12% (w/v) Sodium Dodecyl Sulphate (SDS), 30% (v/v) glycerol, 0.15 M Tris-HCl (pH 6.8), 0.001% (w/v) Brilliant Blue R, 30% (v/v) β-mercaptoethanol] and heated for 5 min. Samples were loaded into a 10% Mini-PROTEAN TGX precast protein gel submerged in 1X Tris/Glycine/SDS buffer (25 mM Tris, 192 mM glycine, 0.1% SDS, pH 8.3), then submitted to vertical transfer in a tetra vertical electrophoresis cell (Bio-Rad, CA, USA) using constant voltage of 150V for 45 min. After protein separation, semi-dry transfer was performed at 15V for 2 h using a BioRad Trans-blot semidry blotter. Membranes were blocked with 5% bovine serum albumin (BSA) for 1 h and incubated with primary antibodies against VASP (Ser239) or GAPDH at 1:1,000 v/v dilution overnight. After washing the primary antibody off, Alexa-488 conjugated phalloidin secondary antibody was incubated for 1 h at room temperature at 1:4,000 v/v dilution. Membranes were visualized using a Typhoon imaging system (GE Healthcare, Hatfield, UK).

### Statistical Analysis

Statistical analyses were obtained from GraphPad Prism 6.0 software (GraphPad Software, San Diego, USA). Quantitative results were expressed as mean ± SEM and individual points for all bar graphs, in order to improve transparency on the variability of data. Sample size varied from three to six independent repeats. Statistical analysis was performed through paired one-way ANOVA and Tukey as post-test with level of significance of 5%.

## Results

### PESc Inhibited Platelet Activation and Aggregation Induced by Different Agonists

**Figure 1:**
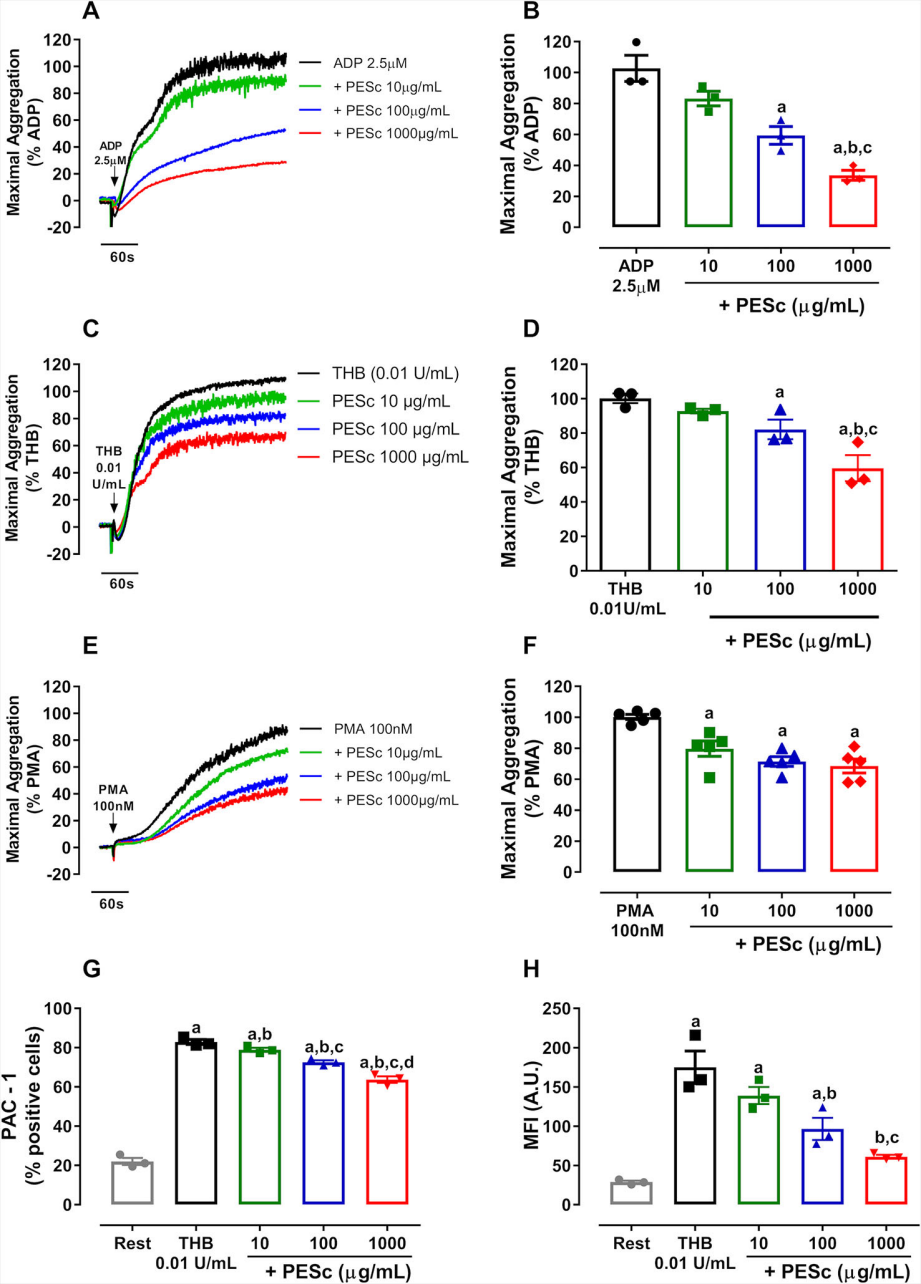
PESc inhibits platelet aggregation and integrin αIIbβ3 activation. Platelet-rich plasma was pretreated with PESc (10, 100, or 1,000 μg/ml) for 25 min and stimulated with ADP **(A)**, thrombin (THB, **C**), or PMA **(E)**. Quantified data is shown next to representative curves for ADP **(B)**, thrombin **(D)**, and PMA **(F)** stimulated platelet aggregation. Washed platelets were pre-treated with PESc under the same conditions, stimulated with thrombin, and incubated with PAC-1 antibody to measure integrin activation. **(G)** Percentage of PAC-1 positive events. **(H)** Mean fluorescence intensity (MFI) of events. a p < 0.05 *vs* first column of graph. b p < 0.05 *vs* second column of graph. c p < 0.05 *vs* third column of graph, d p < 0.05 *vs* fourth column of graph. Data analyzed by paired one-way ANOVA and Tukey as post-test. All bar graphs represent mean ± SEM and individual data points of at least three independent experiments. Arrows indicate when agonists were added.

The initial approach focused on whether PESc would inhibit platelet aggregation. Therefore, different concentrations of PESc were incubated with PRP and platelets were activated with various agonists. The composition of the batch of PESc used in this study is consistent with previously reported (Chagas et al., 2018) (**Supplementary Figure 1**). **Figure 1** displays the inhibitory activity of PESc in ADP-, thrombin-, and PMA-induced platelet aggregation—the strongest inhibition was seen when using ADP, in which PESc promoted a 60% decrease in platelet aggregation. Increased agonist concentration partially overcame the inhibition seen in ADP- and thrombin-induced platelet aggregation (**Supplementary Figure 2**). This persuaded us to use the non-biological agonist PMA, a direct activator of protein kinase C (PKC), as a way to avoid the initial signaling processes triggered by these agonists. Interestingly, concentrations as low as 10 μg/ml of PESc were able to mitigate platelet aggregation induced by PMA by 20%.

Given that PESc inhibited platelet aggregation induced by different agonists, we hypothesized that this extract would also affect integrin αIIbβ_3_ activation. Therefore, we incubated WP with PESc at different concentrations and used PAC-1 antibody to detect active integrin αIIbβ_3_ by flow cytometry. Upon stimulation with thrombin, a six-fold increase in PAC-1 binding was observed and the percentage of positive events increased from 20 to 82% (**Figures 1G, H**). Interestingly, PESc was able to decrease PAC-1 median fluorescence intensity compared to vehicle at concentrations as low as 10 μg/ml (~20% inhibition), reaching 65% inhibition at 1,000 μg/ml (**Figure 1H**). Overall, our data reinforce the strong antiplatelet effects of PESc, due to the significant inhibition observed at concentrations as low as 10 μg/ml, possibly through reacting with molecules that orchestrate integrin αIIbβ_3_ activation.

### Myricetin Was More Potent Than Gallic Acid in Inhibiting Platelet Aggregation

**Figure 2:**
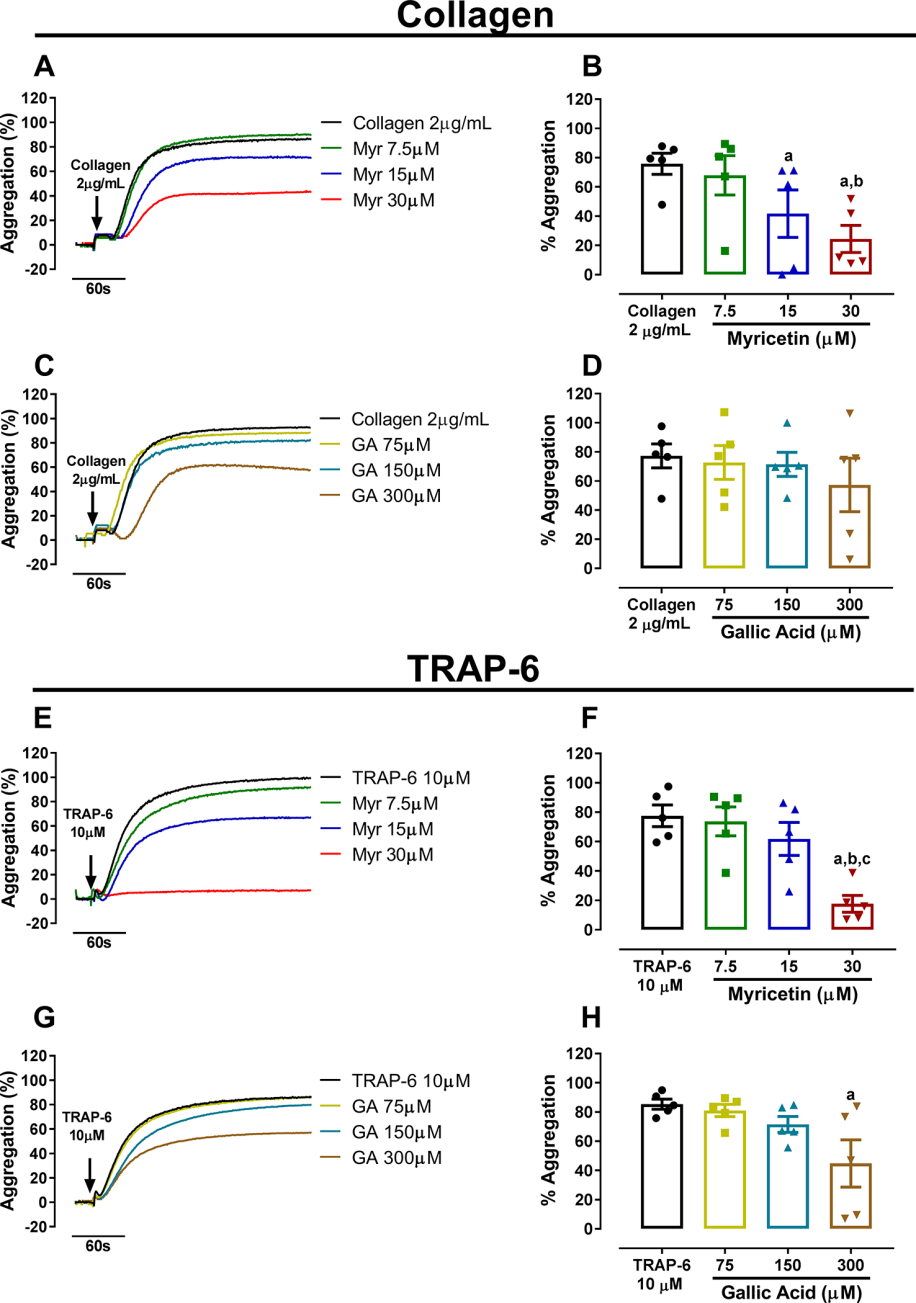
Myricetin inhibits platelet aggregation more potently than gallic acid. Washed platelets (WP) were pre-treated with myricetin (Myr) or gallic acid (GA) for 10 min and stimulated with collagen or TRAP-6. **(A, B)** WP treated with Myr and stimulated with collagen. **(C, D)** WP treated with GA and stimulated with collagen. **(E, F)** WP treated with Myr and stimulated with TRAP-6. **(G, H)** WP treated with GA and stimulated with TRAP-6. Quantified data is shown right next to representative curves. a p < 0.05 *vs* first column of graph. b p < 0.05 *vs* second column of graph. c p < 0.05 *vs* third column of graph. Data analyzed by paired one-way ANOVA and Tukey as post-test. All bar graphs represent mean ± SEM and individual data points of at least three independent experiments. Arrows indicate when agonists were added.

After establishing the antiplatelet potential of PESc and identifying its main components, we investigated the effects of myricetin (most abundant compound) and gallic acid (second most abundant compound) on platelet aggregation. Both PRP (**Supplementary Figure 3**) and WP (**Figure 2**) were incubated with different concentrations of either myricetin or gallic acid and platelets were stimulated with collagen or the thrombin receptor agonist TRAP-6. Myricetin at the highest concentration tested (30 μM) was able to substantially inhibit platelet aggregation induced by both agonists (~80% inhibition for collagen and ~60% inhibition for TRAP-6; **Figures 2B, F**). Gallic acid was unable to inhibit platelet aggregation, except at the higher concentration used (300 μM) in TRAP-6 activated platelets (**Figure 2H**)—an effect that is likely due to cytotoxicity of such high concentration. In fact, gallic acid has been shown to be cytotoxic to different cell lines at concentrations above 50 μM (Park et al., 2008). The effect of myricetin and gallic acid on platelet aggregation should not be compared with data on PESc as different agonists were used. Altogether, these data indicate that myricetin is a potent inhibitor of platelet aggregation at physiologically relevant concentrations, whereas gallic acid yields no inhibitory effect.

### Myricetin Inhibited Platelet Activation and Alpha-Granule Secretion Induced by Different Agonists

**Figure 3:**
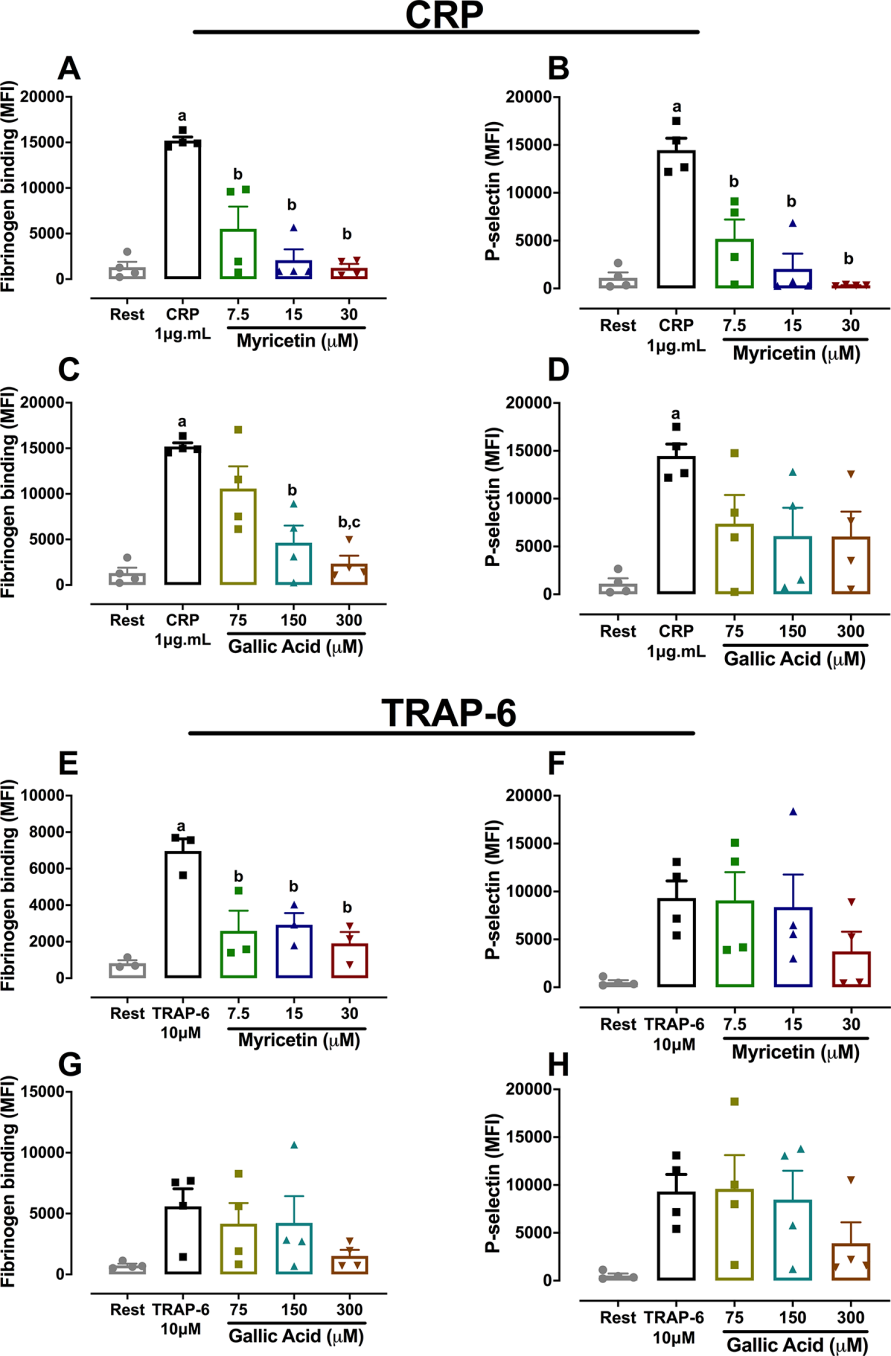
Platelet activation and alpha-granule secretion is inhibited by myricetin but not by gallic acid. Washed platelets (WP) were pre-treated with myricetin or GA for 10 min, stimulated with agonists, and incubated with FITC-coupled fibrinogen and PE/PerCP anti-P-selectin antibodies. Fibrinogen binding **(A)** and P-selectin exposure **(B)** of CRP-activated platelets treated with myricetin. Fibrinogen binding **(C)** or Pselectin exposure **(D)** of CRP-activated platelets treated with GA. Fibrinogen binding **(E)** and P-selectin exposure **(F)** of TRAP-6-activated platelets treated with myricetin. Fibrinogen binding **(G)** and P-selectin exposure **(H)** of TRAP-6-activated platelets treated with GA. a p < 0.05 *vs* first column of graph. b p < 0.05 *vs* second column of graph. c p < 0.05 *vs* third column of graph. Data analyzed by paired one-way ANOVA and Tukey as post-test. All bar graphs represent mean ± SEM as well as individual data points of at least three independent experiments.

Further studies were conducted to assess the effect of both myricetin and gallic acid in platelet function through flow cytometry. Results in **Figure 3** show that myricetin at 15 μM was able to abolish fibrinogen binding and alpha-granule secretion induced by CRP (**Figures 3A, B**). In contrast, gallic acid was only able to reduce fibrinogen binding when incubated at 150 μM, consistent with the limited potency of this phenolic compound to inhibit platelet aggregation (**Figures 3C, D**). When TRAP-6 was used as an agonist, myricetin still inhibited fibrinogen binding, whereas no effect was seen for P-selectin exposure (**Figures 3E, F**). Overall, data herein described suggest myricetin is a flavonoid with potent antiplatelet effects, whereas gallic acid only had an effect at 10× higher concentrations. Therefore, focus was given to myricetin in order to further explore its antiplatelet effects and elucidate possible mechanisms of action.

### Myricetin Inhibits Platelet Adhesion to Collagen and Thrombus Formation Under Flow

**Figure 4:**
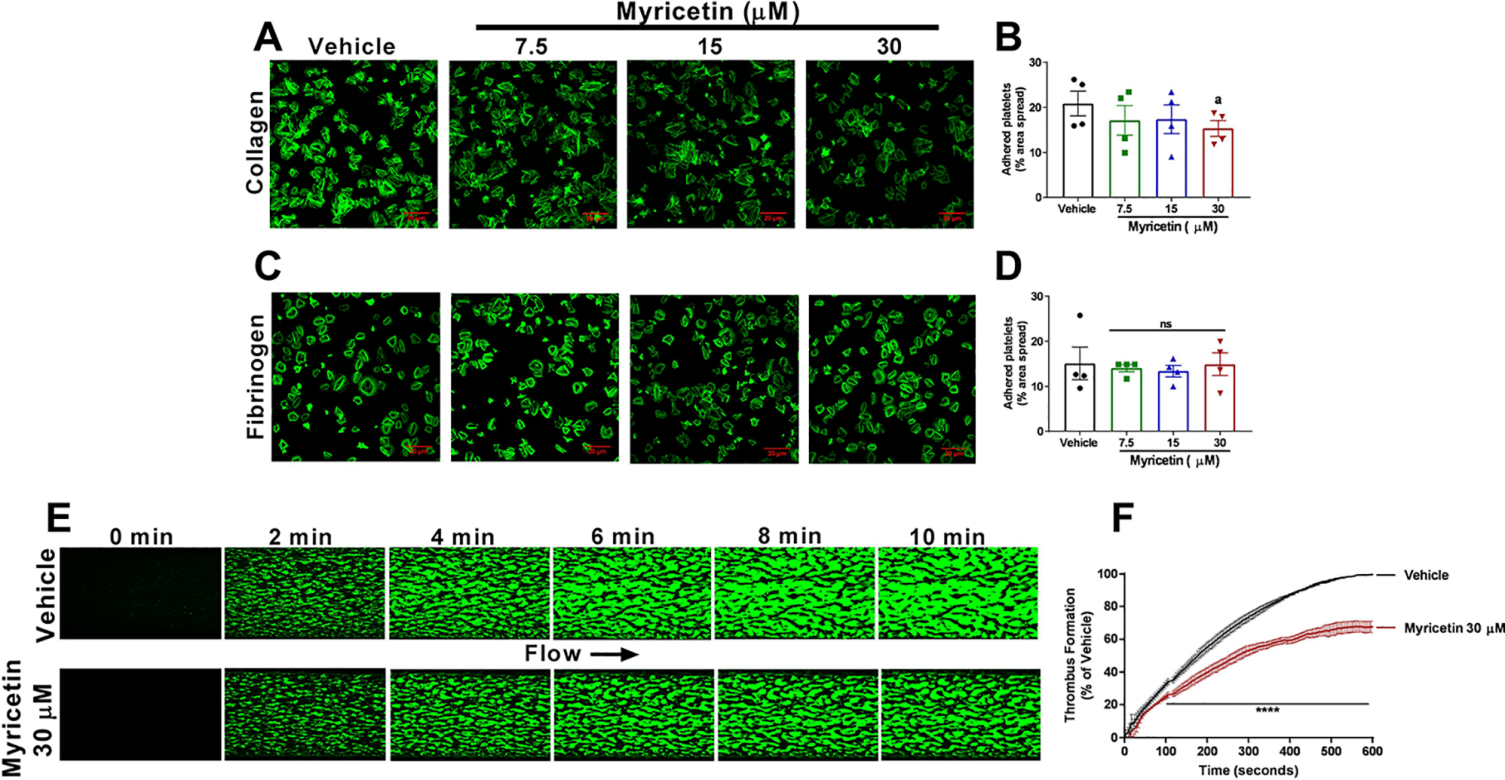
Myricetin inhibits adhesion to collagen and thrombus formation *in vitro*. Washed platelets (WP) were pre-treated with myricetin (7.5, 15, and 30 mM) for 10 min and left to adhere to coverslips coated with 100 mg/ml of collagen **(A**, **B)** or fibrinogen **(C**, **D)** for 45 min. Platelets were stained with Alexa Fluor 488 or 647-conjugated phalloidin for visualization. DioC6-labeled whole blood was pre-treated with myricetin (30 mM) or vehicle control and blood perfused through collagen-coated Vena8 biochip channels at a shear rate of 45 dyn/cm2 for 10 min. **(E)** Representative image of thrombus formation assay. **(F)** Quantification of fluorescence normalized by vehicle control. a p < 0.05 *vs* Vehicle. ****p < 0.001 *vs* Vehicle. ns, non significative. For adhesion assay, bar graphs **(B**, **D)** represent mean ± SEM and individual data points of four independent experiments. For thrombus formation assay, line graph **(F)** represent mean ± SEM of three independent experiments.

Upon vascular injury, platelets start to adhere to components of the sub-endothelium, such as collagen and fibrinogen (Ghoshal and Bhattacharyya, 2014). In order to assess the effect of myricetin on platelet adhesion, WP were left to adhere to collagen or fibrinogen-coated coverslips in the presence or absence of different concentrations of myricetin. The area of platelets spread and representative images of the assay are shown in **Figure 4**. It was clear that myricetin decreased platelet spreading to collagen (~25% inhibition at 30 μM, **Figure 4B**), whereas no effect was seen on platelet spreading to fibrinogen (**Figure 4C**). This is similar to a previous report using PDI-deficient murine platelets (Chang et al., 2012).

Given the antiplatelet effects and inhibition of adhesion to collagen exerted by myricetin, we hypothesized this flavonoid could impact thrombus formation. Therefore, blood was perfused under physiological arterial shear rate into collagen-coated Vena8 biochips as shown in **Figures 4E, F**. It was evident that myricetin treatment decreased thrombus formation (measured as an increase in fluorescence intensity) within the first 100 s, persisting throughout the 10-min assay. These data are consistent with the platelet inhibition herein described for myricetin and expands the importance of this flavonoid to modulate thrombus formation.

### Myricetin Does Not Affect Hemostasis *In Vivo*

**Figure 5:**
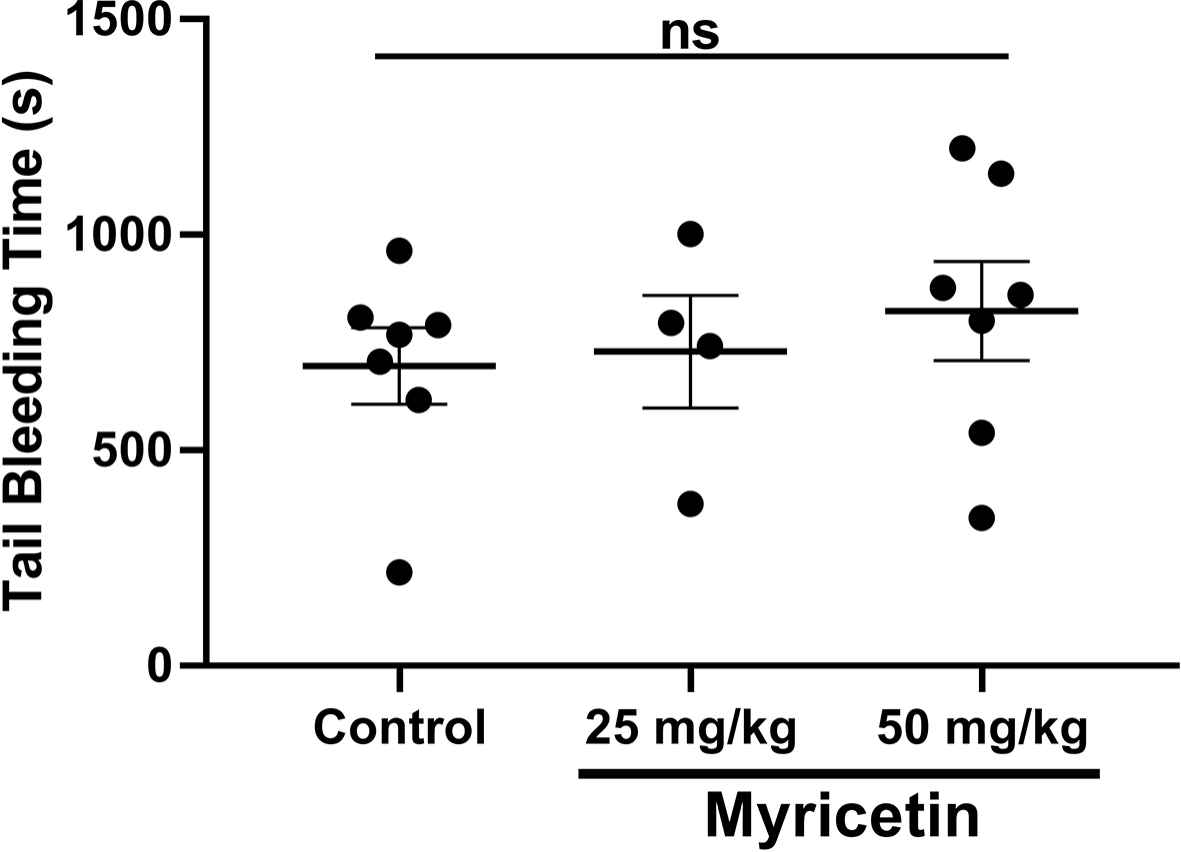
Myricetin does not affect hemostasis *in vivo*. Myricetin at 25 mg/kg or 50 mg/kg as well as vehicle control were administered to healthy mice by oral gavage for three consecutive days. Tail bleeding was measured after tail tip amputation. Data expressed as mean ± SEM as well as individual points. There was no statistical difference between groups.

After establishing antiplatelet and anti-thrombotic properties for myricetin, we then tested its impact on hemostasis. Healthy mice (10–12 weeks of age) were given myricetin (25 or 50 mg/kg) orally for 3 consecutive days, upon which bleeding time was measured after tail tip removal. Results are shown in **Figure 5**. Mice treated with myricetin displayed similar bleeding time when compared to vehicle control. Notably Kim et al. (2013) have reported that genetic deletion of PDI in platelets is tolerated and bleeding time, similarly unaffected. Of note, myricetin did not induce VASP phosphorylation (**Supplementary Figure 4**).

Therefore, considering that 1) myricetin inhibited platelet aggregation induced by different agonists, 2) that PDI is a key modulator of integrin αIIbβ_3_ function located at the end of the platelet activation pathway, 3) that myricetin inhibited platelet spreading on collagen but not on fibrinogen, similar to a PDI knockout model (Kim et al., 2013) 4) that myricetin showed no effect on hemostasis, also comparable to a platelet-specific PDI knockout model (Kim et al., 2013), and 5) that some flavonoids have been described as PDI inhibitors (Jasuja et al., 2012; Giamogante et al., 2018), we decided to investigate the biochemical effects of myricetin on PDI and other thiol isomerases important for platelet function.

### Myricetin Binds Close to the Active Redox Sites of PDI, ERp5, ERp57, and ERp72

The possible interaction between myricetin and PDI, ERp5, ERp57, and ERp72 was initially assessed through quenching of the fluorescence emitted by tryptophan residues exposed near the redox active site WCGHC, as described for ERp57 (Trnkova et al., 2013). **Table 1** shows the values for the Stern-Volmer constant *K*_SV_, the quencher coefficient *K*q, the binding constant *K*_b_, and the dissociation constant *K*_d_ for each protein studied. Values for *K*_SV_ and *K*q were within the same order of magnitude for all of the thiol isomerases tested, indicating a similar binding affinity between these proteins and myricetin (**Table 1**). Representative quenching curves for each protein and Stern-Volmer plot are shown in **Supplementary Figure 5**. Notwithstanding, it is possible for a compound to bind to thiol isomerases without affecting their function, as previously described for the interaction between ellagic acid and ERp57 (Giamogante et al., 2018). Therefore, the ability of myricetin to inhibit the reductase activity of these thiol isomerases was explored.

**Table 1:** Constants calculations based on protein quenching studies.

	*K*_SV_(M^-1^)	*K*q (M^-1^s^-1^)	*K*_b_ (M^-1^)	*K*_d_ (M)
ERp5	48,750 ± 9,554	4.87 · 10^12^	5.72 · 10^5^	1.74 · 10^-6^
ERp72	27,515 ± 5,675	2.75 · 10^12^	3.94 · 10^5^	2.53 · 10^-6^
ERp57	29,777 ± 5,966	2.97 · 10^12^	5.44 · 10^5^	1.83 · 10^-6^
PDI	21,207 ± 0,877	2.12 · 10^12^	2.34 · 10^5^	4.26 · 10^-6^

K_SV_: Stern-Volmer constant. Kq: Quenching rate constant. K_b_: Binding constant.

K_d_: Dissociation constant. Data presented as Mean ± SEM.

### Myricetin Inhibits the Reductase Activity of PDI and Erp5

**Figure 6:**
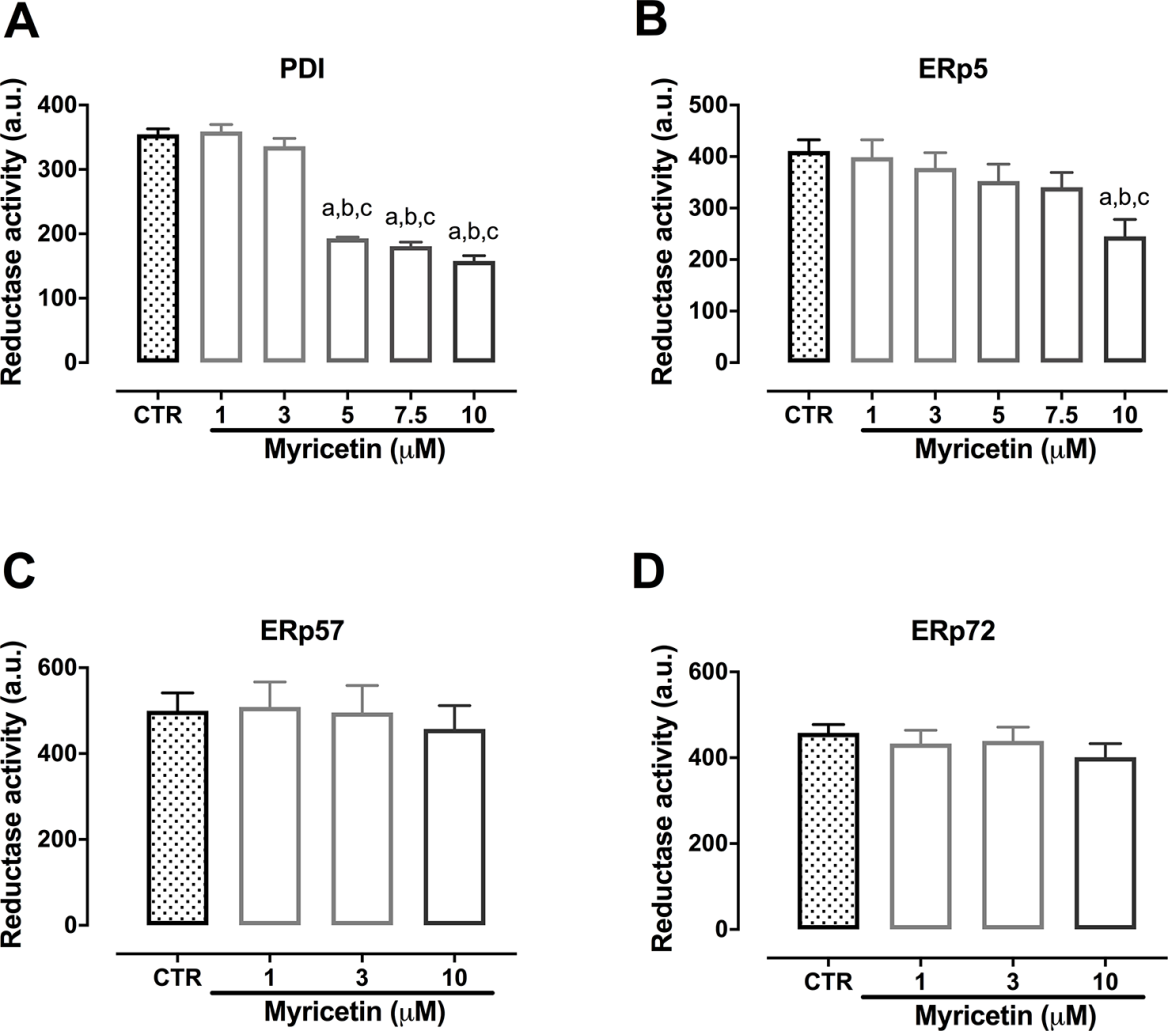
Myricetin inhibits reductase activity of PDI and ERp5. Recombinant proteins were incubated with myricetin (0.01 to 10 μM) in a black 96-wells plate for 10 min followed by addition of DTT (5 μM) and Di-E-GssG (200 nM). Fluorescence was read every 30 s for 30 min. Final point fluorescence at 30 min is shown for ERp5 **(A**, **B)**, PDI **(C**, **D)**, ERp72 **(E**, **F)**, and ERp57 **(G**, **H)**. Data represent at least three independent experiments run at least in duplicate and error bars indicate SEM. a p < 0.05 *vs* first column of graph. b p < 0.05 *vs* second column of graph. c p < 0.05 *vs* third column of graph.

The highly sensitive fluorescent probe Di-E-GssG was used to assess the reductase activity of thiol isomerase in the presence or absence of myricetin. Results shown in **Figure 6** demonstrate the ability of myricetin to inhibit both PDI and ERp5, being more potent against PDI (**Figures 6A, B**). On the other hand, myricetin was unable to inhibit the reductase activity of ERp57 or ERp72 at the concentrations tested (**Figures 6C, D**). Therefore, we proceeded to investigate possible binding mechanisms between myricetin and PDI and ERp5 using a molecular docking approach.

### Myricetin Is Predicted to Form Non-Covalent Bonds Close to the Active Redox Sites of PDI and ERp5

**Figure 7:**
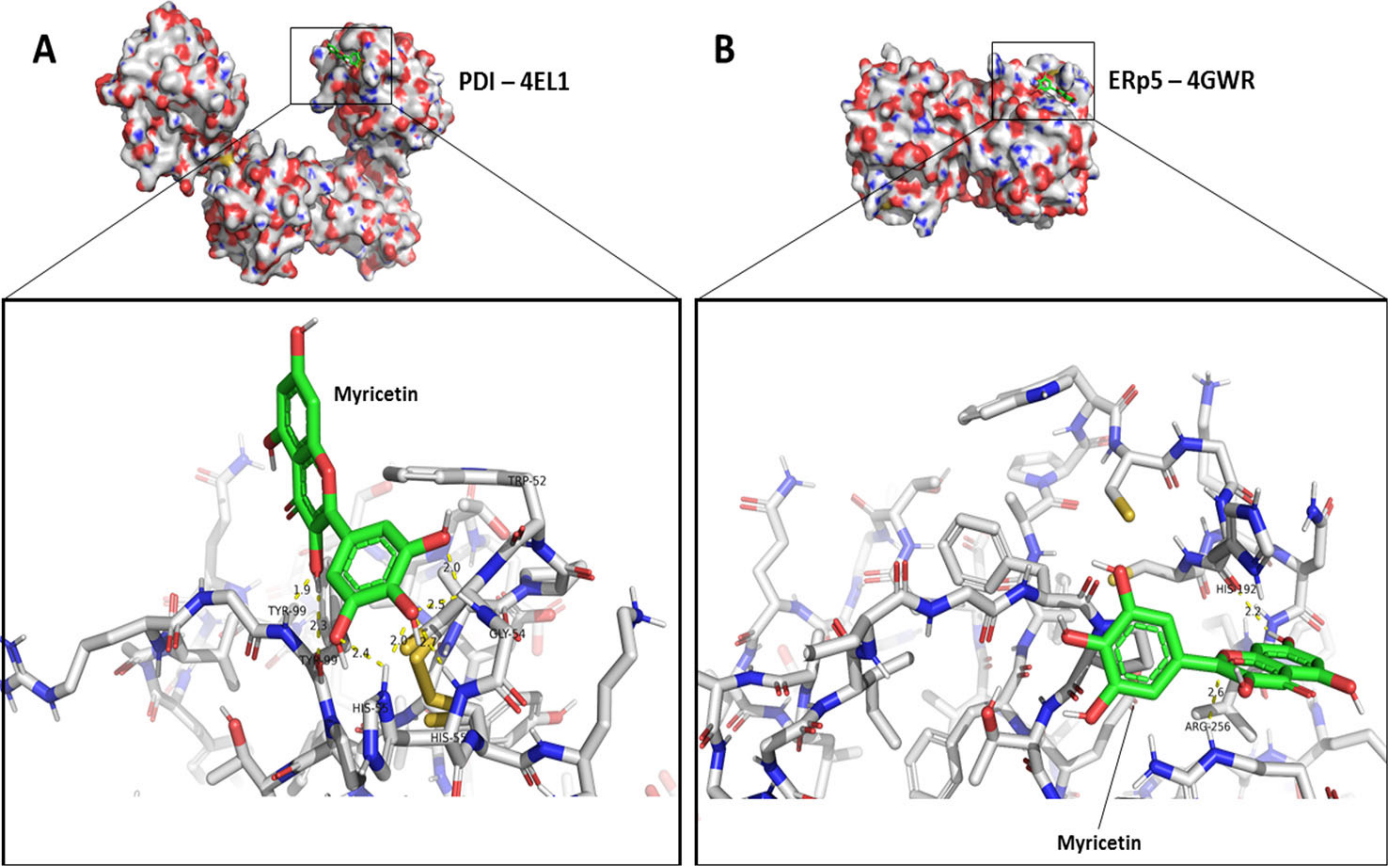
Feasible interactions for Myricetin with PDI and ERp5 predicted through molecular docking. The predicted poses of interaction between myricetin and different thiol isomerases were assessed using AutoDock 4.2 package as described in Methods. **(A)** Pose of highest affinity for PDI and detailed intermolecular interactions. **(B)** Pose of highest affinity for ERp5 and detailed intermolecular interactions. Additional information on interactions and other possible poses are described in **Supplementary Tables 1** and **2**.

To assess the nature of the interaction between myricetin and thiol isomerases ERp5 and PDI, *in silico* experiments using molecular docking were conducted. Since the protein quenching studies suggested an interaction between myricetin and the Trp residues of the thiol isomerases, the grid box of analysis for molecular docking was centered at Trp_189_ for ERp5 and Trp_52_ for PDI, which are near the active site of each protein. Results shown in **Figure 7** provide an overview of the interactions found for the pose of highest affinity between ligand and protein, whereas the full description of interactions can be accessed on **Supplementary Tables 1** and **2**. It is notable that myricetin displayed similar affinity to both PDI and ERp5 (**Figure 7**), which corroborates *in vitro* findings (**Table 1**). Likewise, all of the interactions herein described are non-covalent bonds, with hydrogen bonding constituting the vast majority of these, even though it is possible for myricetin to form adducts with thiols through carbons 2’ and 6’ on ring B (Masuda et al., 2013).

## Discussion

This study expands the applicability of PESc and myricetin, a flavonoid of widespread occurrence among plants and the most abundant in PESc, on platelet function and thrombus formation. Additionally, it offers a novel mechanism by which this flavonoid may inhibit platelets and thrombus formation. Mechanistically, it was shown that myricetin was able to bind to thiol isomerases and inhibit the reductase activity of PDI and ERp5 possibly due to non-covalent bonds between the compound and amino acids adjacent to the redox active site of these proteins.

We first showed that PESc was able to inhibit platelet function. PESc also inhibited platelet aggregation induced by PKC activator PMA (a phorbol ester that directly activates PKC), which suggests some compounds were able to cross the platelet cell membrane, probably targeting PKC or downstream molecules, i.e. signaling that occurs at the end of the platelet activation pathway. Moreover, the inhibition of platelet function herein described for PESc is in accordance with reports showing that a green tea flavonoid-rich extract reduced platelet aggregation and integrin αIIbβ_3_ activation upon stimulus with ADP, thrombin, or collagen (Kang et al., 2001). Given that myricetin and gallic acid were the two most abundant polyphenols found within PESc, we then proceeded to test these compounds individually.

Myricetin inhibited platelet aggregation and activation induced by agonists of the collagen and thrombin pathways, whereas gallic acid showed little to no effect even at 10× higher concentrations. This is in agreement with previous reports showing that myricetin strongly inhibited collagen- (Dutta-Roy et al., 1999) and arachidonic acid-evoked platelet aggregation (Lescano et al., 2018). Interestingly this latter work reported that myricetin does not inhibit cyclooxygenase activity in platelets (Lescano et al., 2018). It has been described that gallic acid is able to inhibit platelet aggregation only at exceedingly high concentrations (Chang et al., 2012) which is corroborated by our data showing no effect below 300 μM. In addition, (Dang et al., 2014) showed that myricetin was able to reach a peak plasma concentration of 10 μM upon a single oral dose of 100 mg/kg in rats, corroborating that the concentrations tested in our study could be achievable *in vivo*.

The effect of myricetin on platelet activation is also compatible with that previously shown for quercetin, a flavonoid of similar structure. Navarro-Nuñez et al. (2009) reported that quercetin was able to inhibit platelet aggregation and signaling induced by thrombin and specific agonists of the thrombin receptors protease-activated receptor 1 (PAR1) and 4 (PAR4). The ability of myricetin to inhibit platelet aggregation and activation induced by different agonists suggests this flavonoid may act on molecules common to each pathway. The lack of effect of gallic acid on platelet function, coupled with the strong inhibition exerted by myricetin prompted us to focus on myricetin to further assess its mechanisms of action.

Some flavonoids, such as nobiletin, have been shown to increase VASP phosphorylation (Jayakumar et al., 2017), which is a key inhibitory molecule in platelets. Myricetin did not induce the phosphorylation of VASP at Ser_239_ (**Supplementary Figure 4**), suggesting this is probably not the target for this flavonoid. Likewise, quercetin has been shown to bind to the Thromboxane A_2_ (TxA_2_) receptor (TPR) and this could also be a potential mechanism of action for myricetin. However, previous literature has shown that SQ-29548, a specific TPR inhibitor, displayed little effect on CRP-induced platelet activation (Taylor et al., 2014) and that platelet aggregation induced by CRP was independent of TxA_2_ (Jarvis et al., 2002). In addition, TRAP-induced aggregation was found to be aspirin-insensitive, suggesting a minor role for TxA_2_ (Chung et al., 2002). Therefore, although the effects of Myricetin on TxA_2_ pathway cannot be excluded, we argue that this is likely not the main target of this flavonoid, since it was able to potently inhibit platelet aggregation and activation induced by CRP and TRAP-6.

Platelets express two principal membrane receptors that are able to bind collagen: GPVI and integrin α_2_β_1_. Despite GPVI being considered the primary collagen receptor involved in platelet aggregation and activation (Kehrel et al., 1998), integrin α2β1 was shown to be the main platelet adhesive receptor to collagen (Inoue et al., 2003). Moreover, it was recently shown that GPVI could also bind and contribute to platelet adhesion and spreading to immobilized fibrinogen (Mangin et al., 2018), suggesting GPVI is unlikely to be a target for myricetin since this flavonoid did not inhibit platelet spreading to fibrinogen (**Figure 4C**). Interestingly, it was demonstrated that integrin α_2_β_1_ is in close proximity and is regulated by PDI (Lahav et al., 2003). In fact, platelet-specific PDI-deficient mice were unable to form proper thrombi on collagen-coated surfaces, even though their platelets spread normally on fibrinogen (Kim et al., 2013), similar to myricetin (**Figure 4**). This same report described no changes in bleeding time between wildtype and platelet-PDI deficient mice, also in accordance with data herein described for myricetin. Therefore, we decided to assess the interaction between myricetin and thiol isomerases.

Initially, thiol isomerases were incubated with myricetin and changes in tryptophan fluorescence were measured. Protein quenching studies showed that myricetin was able to bind to all of the thiol isomerases tested. Values of the dissociation constant *K*q greater than 2.0 × 10^10^ M^-1^s^-1^ support the formation of complexes between quencher and protein (Ware, 1962), suggesting myricetin forms a complex or complexes with the thiol isomerases tested. Interestingly, the *K*q herein reported for myricetin is one order of magnitude lower than that described for other flavonoids binding to ERp57 (Giamogante et al., 2016). Considering that the dissociation constant is inversely related to binding affinity, these results suggest myricetin has a high binding affinity to thiol isomerases, at the μM range. These results, however, do not allow the conclusion of whether the interaction between myricetin and thiol isomerases is due to static or dynamic binding.

Despite being able to bind to PDI, ERp5, ERp57, and ERp72, myricetin was only able to inhibit the reductase activity of PDI and ERp5 at concentrations able to be biologically reached. Quercetin, a structurally similar flavonoid was reported to be a weak inhibitor of PDI (Jasuja et al., 2012), whereas quercetin derivatives, such as isoquercetin (Stopa et al., 2017) and rutin (Jasuja et al., 2012), were shown to be potent inhibitors of PDI reductase activity. It is important to note that we used the fluorescent EGSH method whereas these reports used insulin turbidimetry to assess reductase activity. Thus, differing results would be anticipated since the fluorescent EGSH method is considered to be more sensitive than insulin turbidimetry (Raturi and Mutus, 2007). This is corroborated by a recent report showing distinct behavior of a new class of PDI inhibitors tested in both assays (Bekendam et al., 2016). Nonetheless, the inhibition exerted by myricetin is comparable to that of the flavonoid punicalagin, a non-competitive inhibitor of ERp57 (Giamogante et al., 2018).

Molecular docking studies predicted interactions between myricetin and amino acids adjacent to the tryptophan residues near the redox active sites in each thiol isomerase. This indicates that the possible quenching mechanism is unlikely to be a direct complex between ligand and tryptophan. One possibility is that the binding of myricetin to adjacent amino acids such as Tyr_99_ of PDI or His_192_ of ERp5, may induce excited-state proton or electron transfer from these amino acids to the Trp nearby, which would quench its fluorescence, as previously described (Chen and Barkley, 1998). The lack of covalent bonds predicted for myricetin and thiol isomerases suggest a weak and reversible interaction, similar to that described for rutin and PDI (Wang et al., 2018), which makes it more difficult to assess such interaction *in vitro*. Since myricetin was predicted to bind close to the redox CGHC site of PDI and ERp5, it is also hypothesized that myricetin inhibits the reductase activity through an allosteric effect, similar to what described for rutin (Wang et al., 2018). Future studies are needed to confirm these findings.

In conclusion, this study expands the applicability of PESc as an extract, and describes myricetin as a novel inhibitor of thiol isomerases ERp5 and PDI with potent antiplatelet and anti-thrombotic properties. Moreover, myricetin was shown to have no effect on hemostasis, initially suggesting lower chances of bleeding upon myricetin treatment. Nevertheless, future studies with longer treatment regimens are needed to further assess the safety and efficacy of this flavonoid, as well as the interaction of myricetin with other proteins, such as thioredoxin reductase (Lu et al., 2006) and kinases (Navarro-Nuñez et al., 2009). Therefore, this study may offer new insights into the complementary use of myricetin for the treatment of thrombosis, corroborating the promising use of flavonoids to treat cardiovascular diseases with thrombotic outcomes.

## Data Availability Statement

The datasets generated for this study are available on request to the corresponding authors.

## Ethics Statement

The studies involving human participants were reviewed and approved by the Research Ethics Committees of the Federal University of Maranhão and University of Reading. The patients/participants provided their written informed consent to participate in this study. The animal study was reviewed and approved by the National Council for the Control of Animal Experimentation (CONCEA, Brazil) and the local Animal Care and Welfare Committee of the Federal University of Maranhao, under ruling number 23115.018725/2017-19.

## Author Contributions

RG designed the study, performed experiments, analyzed data, and drafted the manuscript. SS, JS, JF, HS, and VC performed experiments and analyzed data. SA generated recombinant proteins used in experiments. AT supervised experiments, discussed data, and reviewed the manuscript. JG discussed data and reviewed the manuscript. AP designed the study, discussed the data, drafted and reviewed the manuscript.

## Funding

This study was funded by the British Heart Foundation (RG/15/2/31224), Medical Research Council (MR/J002666/1), Coordenação de Aperfeiçoamento de Pessoal de Nível Superior – Brasil (CAPES) – Finance Code 001, and Fundação de Amparo à Pesquisa e ao Desenvolvimento Científico e Tecnológico do Maranhão, FAPEMA (PAEDT-00376/14, APCINTER 02698/14). AT was supported by CSIC grupos I+D 2014 and 2018 (536) and FAPEMA (PVI-05558/15). AP was supported by FAPEMA (BEEP-02511/18).

## Conflict of Interest

The authors declare that the research was conducted in the absence of any commercial or financial relationships that could be construed as a potential conflict of interest.

The reviewer [MC] declared a past co-authorship with one of the authors [JG] to the handling editor.

## Abbreviations

CRP, collagen-related peptide; GAPDH, glyceraldehyde 3-phosphate dehydrogenase; PAR, protease-activated receptor; PDI, protein disulfide isomerase; PESc, polyphenol-rich extract of *S. cumini* leaf; PKC, protein kinase c; PMA, phorbol-12-myristate-13-acetate; PRP, platelet-rich plasma; *S. cumini, Syzygium cumini* (L.) Skeels; TxA2, thromboxane A_2_; TPR, thromboxane A_2_ receptor; TRAP-6, thrombin receptor activator peptide 6; VASP, vasodilator-stimulated phospho-protein; WP, washed platelets.

## Supplementary Material

Supplementary Figure 1 Chromatographic fingerprint of PESc and flavonoid standards. UV detection of standards for gallic acid, myricetin and quercetin (A) or a sample of PESc (B) were analysed through LC-MS/MS as described in Methods. In addition, each fraction was purified and their identity confirmed by HPLC-MS/MS studies. Peak 1 corresponded to gallic acid, peaks 2 and 3 to myricetin glycoside derivatives, peak 4 to myricetin and peak 5 to quercetin. Structures of the identified compounds are shown in (C).

Supplementary Figure 2 Increased agonist concentration partially overcome anti-platelet effect of PESc. Platelet-rich plasma was pre-treated with PESc (10, 100 or 1000 μg/mL) for 25 minutes and stimulated with ADP (A-D) or thrombin (THB, E-H). Representative traces for 2.5 μM ADP (A) and 5 μM ADP (C). Representative traces for 0.01 U/mL THB (E) and 0.02 U/mL THB (G). Quantified data is shown next to representative curves. a p<0.05 *vs* first column of graph. b p<0.05 *vs* second column of graph. c p<0.05 *vs* third column of graph. Data analysed by paired one-way ANOVA and Tukey as post-test. All bar graphs represent mean ± SEM and individual data points of at least 3 independent experiments. Arrows indicate when agonists were added.

Supplementary Figure 3 Decreased effect of Myricetin in platelet-rich plasma. Platelet-rich plasma (PRP) was pre-treated with myricetin (Myr) or gallic acid (GA) for 10 minutes and stimulated with collagen or TRAP-6. (A) PRP treated with Myr and stimulated with collagen. (C) PRP treated with GA and stimulated with collagen. (E) PRP treated with Myr and stimulated with TRAP-6. (G) PRP treated with GA and stimulated with TRAP-6. Quantified data is shown right next to representative curves. a p<0.05 *vs* first column of graph. b p<0.05 *vs* second column of graph. c p<0.05 *vs* third column of graph. Data analysed by paired one-way ANOVA and Tukey as post-test. All bar graphs represent mean ± SEM and individual data points of at least 3 independent experiments. Arrows indicate when agonists were added.

Supplementary Figure 4 Myricetin does not induce VASP phosphorylation. Resting WP were incubated with myricetin (7.5, 15 and 30 μM) or PAPA-NONOate (100 μM, positive control) for 10 minutes and lysed in laemmli buffer supplemented with reducing agent. Lysed cells were processed as described in Material and Methods and probed for VASP_s239_ and GAPDH as loading control. Bar graph represent present the mean of four independent experiments run and error bars indicate SEM. Data compared using One-way ANOVA followed by Tukey post-test. There were no statistical differences between groups.

Supplementary Figure 5 Myricetin quenches fluorescence of ERp5, ERp57, ERp72 and PDI. Recombinant proteins were incubated with myricetin (0.01 to 10 μM) in a black 96-wells plate for 10 minutes and fluorescence spectra acquired in a fluorimeter using excitation set at 280 nm. Representative fluorescence spectra shown for ERp5 (A), ERp57 (B), ERp72 (C) and PDI (D). (E) Stern-volmer plot of quenching data is shown as the linear regression between F_0_/F and log of myricetin concentration in mM where F_0_ is the fluorescence of vehicle and F is the fluorescence in the presence of increasing concentrations of myricetin. Data represent at least three independent experiments run at least in duplicate and error bars indicate SEM.
