# Supplementary material for: Myricetin, the Main Flavonoid in Syzygium cumini Leaf, Is a Novel Inhibitor of Platelet Thiol Isomerases PDI and ERp5
Source: Front Pharmacol. 2020 Jan 31;10:1678. doi: 10.3389/fphar.2019.01678 (PMC7011086; doi:10.3389/fphar.2019.01678)
Supplement: Supplementary file 1 [file DataSheet_1.docx]

**Supplementary Table 1 Predicted interactions between Myricetin and ERp5 (4GWR) interaction**

| **Pose** | **Myricetin** | **Distance (Å)** | **Intermolecular Interaction** | **Protein** | **Affinity (kcal/mol)** |
| --- | --- | --- | --- | --- | --- |
| 1 | O1 | 2.5 | = O – HN | H192 | -5.3 |
|  | O1 | 2.6 | = O – HN | R256 |  |
|  | H3 | 2.2 | OH – O = | H192 |  |
| 2 | O1 | 2.3 | = O – HN | W189 | -4.8 |
|  | O18 | 2 | = O – HN | Y186 |  |
|  | O21 | 2 | HO – HN | G191 |  |
| 7 | O17 | 2.1 | HO – HN | W189 | -4.6 |
|  | O18 | 2.2 | = O – HN | F237 |  |
|  | O19 | 2.2 | OH – O = | F237 |  |
|  | H6 | 2.2 | OH – O = | W189 |  |
|  | O21 | 1.9 | HO – HN | G191 |  |
|  | H1 | 2.3 | HO – HN | F237 |  |
| 8 | H9 | 2.4 | OH – O = | F237 | -4.4 |
|  | O19 | 2.7 | HO – HN | G191 |  |
|  | O19 | 2.3 | HO – HN | H192 |  |
| 14 | O17 | 2.1 | HO – HN | G191 | -4.2 |
|  | H9 | 2.3 | OH – O = | W189 |  |
|  | O21 | 2.1 | HO – HN | W189 |  |
|  | O22 | 2.2 | HO – HN | F237 |  |
|  | H7 | 2.4 | OH – O = | F237 |  |
| 16 | O22 | 2.4 | HO – HN | F237 | -4.2 |
|  | H7 | 2.1 | OH – O = | F237 |  |
|  | O21 | 2 | HO – HN | W189 |  |
|  | O1 | 2.2 | = O – HN | G191 |  |
|  | O20 | 2.2 | HO – HN | K194 |  |

**Supplementary Table 2 Predicted interactions between Myricetin and PDI (4EL1) interaction**

| **Pose** | **Myricetin** | **Distance (Å)** | **Intermolecular Interaction** | **Protein** | **Affinity (kcal/mol)** |
| --- | --- | --- | --- | --- | --- |
| 1 | O17 | 1.9 | HO – HN | Y99 | -5.5 |
|  | H9 | 2.3 | OH – O = | Y99 |  |
|  | O21 | 2.4 | HO – HN | H55 |  |
|  | O22 | 2 | HO – HN | H55 |  |
|  | O22 | 2.7 | HO – HN | H55 |  |
|  | O22 | 2.5 | HO – HN | G54 |  |
|  | O23 | 2 | HO – HN | G54 |  |
| 3 | O21 | 2.2 | HO – HN | K81 | -5.2 |
|  | O17 | 2.2 | HO – HO | Y49 |  |
|  | H9 | 2 | OH – OH | D83 |  |
| 5 | O19 | 2.4 | HO – HN | K81 | -5.2 |
|  | O18 | 2.4 | = O – HN | K81 |  |
| 7 | O17 | 2.9 | HO – HN | K81 | -5.1 |
|  | H9 | 2.2 | OH – O = | E23 |  |
|  | H9 | 2.9 | OH – OH | E23 |  |
|  | O22 | 2 | HO – HN | K57 |  |
| 11 | O21 | 2 | HO – HN | G54 | -4.9 |
|  | O22 | 2.4 | HO – HN | G54 |  |
|  | O22 | 2.4 | HO – HN | H55 |  |
|  | O22 | 1.9 | HO – HN | H55 |  |
|  | O23 | 2.8 | HO – HN | H55 |  |
|  | O1 – C10 | 4 – 4,2 | π – π | W52 |  |
| 14 | O17 | 2 | HO – HN | G54 | -4.9 |
|  | H7 | 2.4 | OH – O = | Y99 |  |
|  | O22 | 2.4 | HO – HN | Y99 |  |
|  | O21 | 2.3 | HO – HN | Y99 |  |
| 15 | O19 | 2.3 | HO – HN | Q92 | -4.8 |
|  | H3 | 1.9 | OH – O = | A84 |  |
|  | C11 – C16 | 3.7 | π – π | W52 |  |
|  | O21 | 2.4 | HO – HN | Y99 |  |
