# Supplementary figures and images for: Myricetin, the Main Flavonoid in Syzygium cumini Leaf, Is a Novel Inhibitor of Platelet Thiol Isomerases PDI and ERp5
Source: Front Pharmacol. 2020 Jan 31;10:1678. doi: 10.3389/fphar.2019.01678 (PMC7011086; doi:10.3389/fphar.2019.01678)

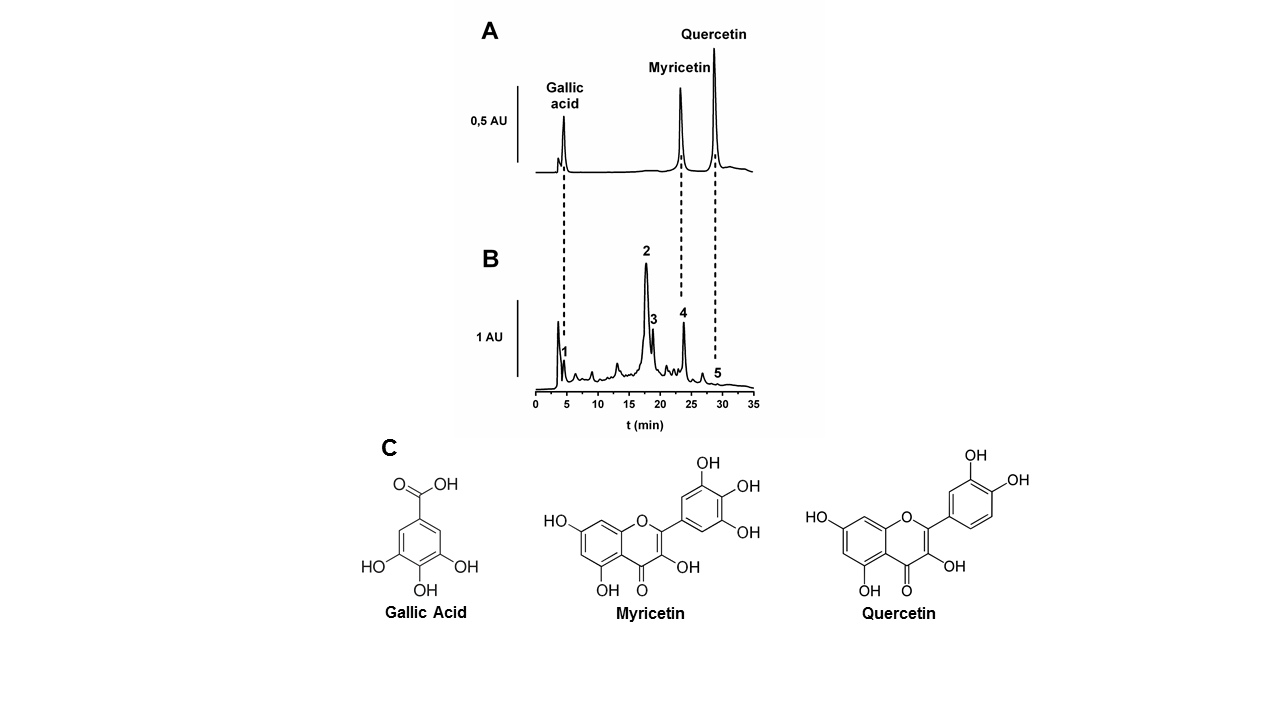

Supplement: Supplementary Figure 1 — Chromatographic fingerprint of PESc and flavonoid standards. UV detection of standards for gallic acid, myricetin and quercetin (A) or a sample of PESc (B) were analysed through LC-MS/MS as described in Methods. In addition, each fraction was purified and their identity confirmed by HPLC-MS/MS studies. Peak 1 corresponded to gallic acid, peaks 2 and 3 to myricetin glycoside derivatives, peak 4 to myricetin and peak 5 to quercetin. Structures of the identified compounds are shown in (C). [file Image_1.tif]

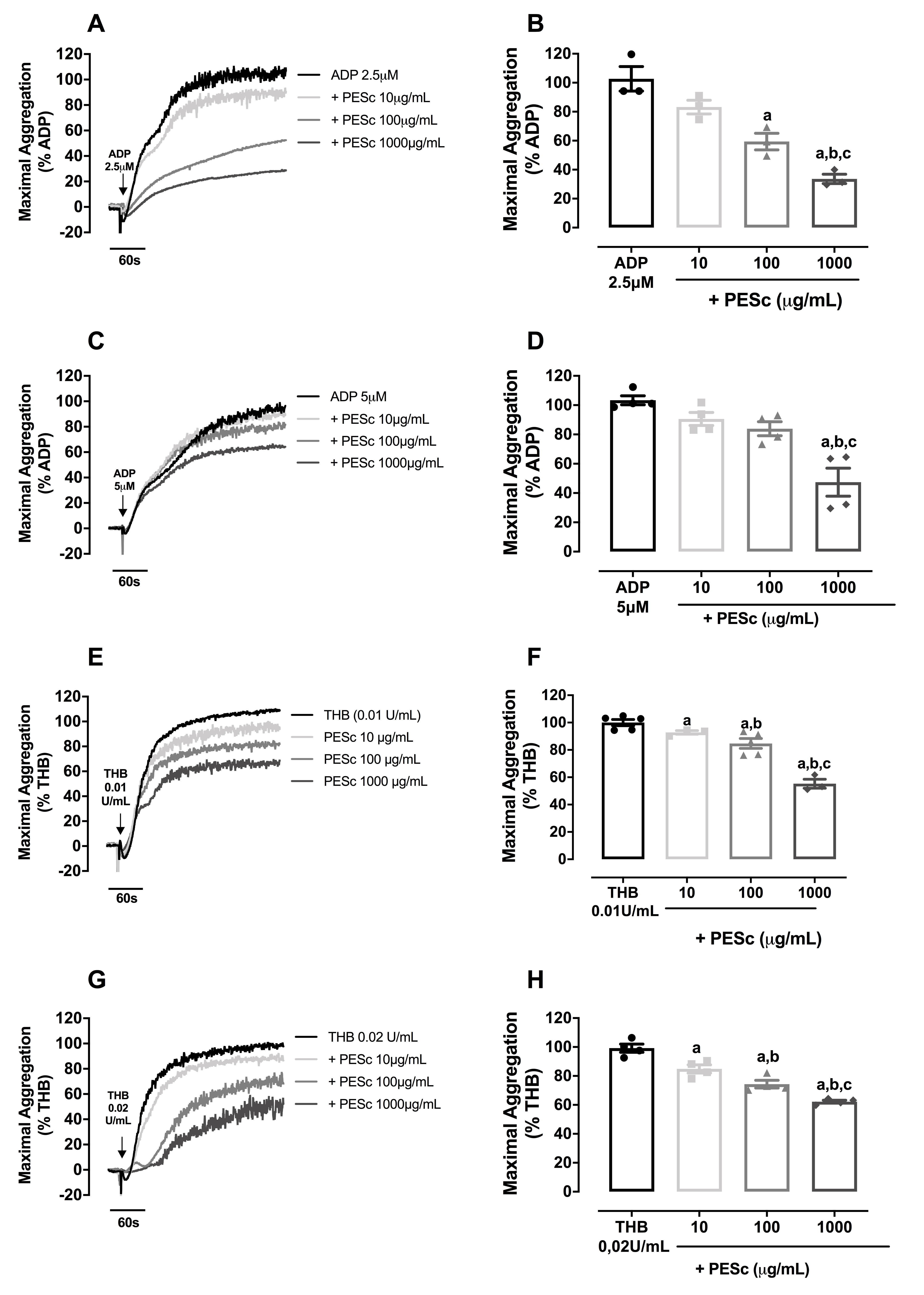

Supplement: Supplementary Figure 2 — Increased agonist concentration partially overcome anti-platelet effect of PESc. Platelet-rich plasma was pre-treated with PESc (10, 100 or 1000 μg/mL) for 25 minutes and stimulated with ADP (A-D) or thrombin (THB, E-H). Representative traces for 2.5 μM ADP (A) and 5 μM ADP (C). Representative traces for 0.01 U/mL THB (E) and 0.02 U/mL THB (G). Quantified data is shown next to representative curves. a p<0.05 vs first column of graph. b p<0.05 vs second column of graph. c p<0.05 vs third column of graph. Data analysed by paired one-way ANOVA and Tukey as post-test. All bar graphs represent mean ± SEM and individual data points of at least 3 independent experiments. Arrows indicate when agonists were added. [file Image_2.tiff]

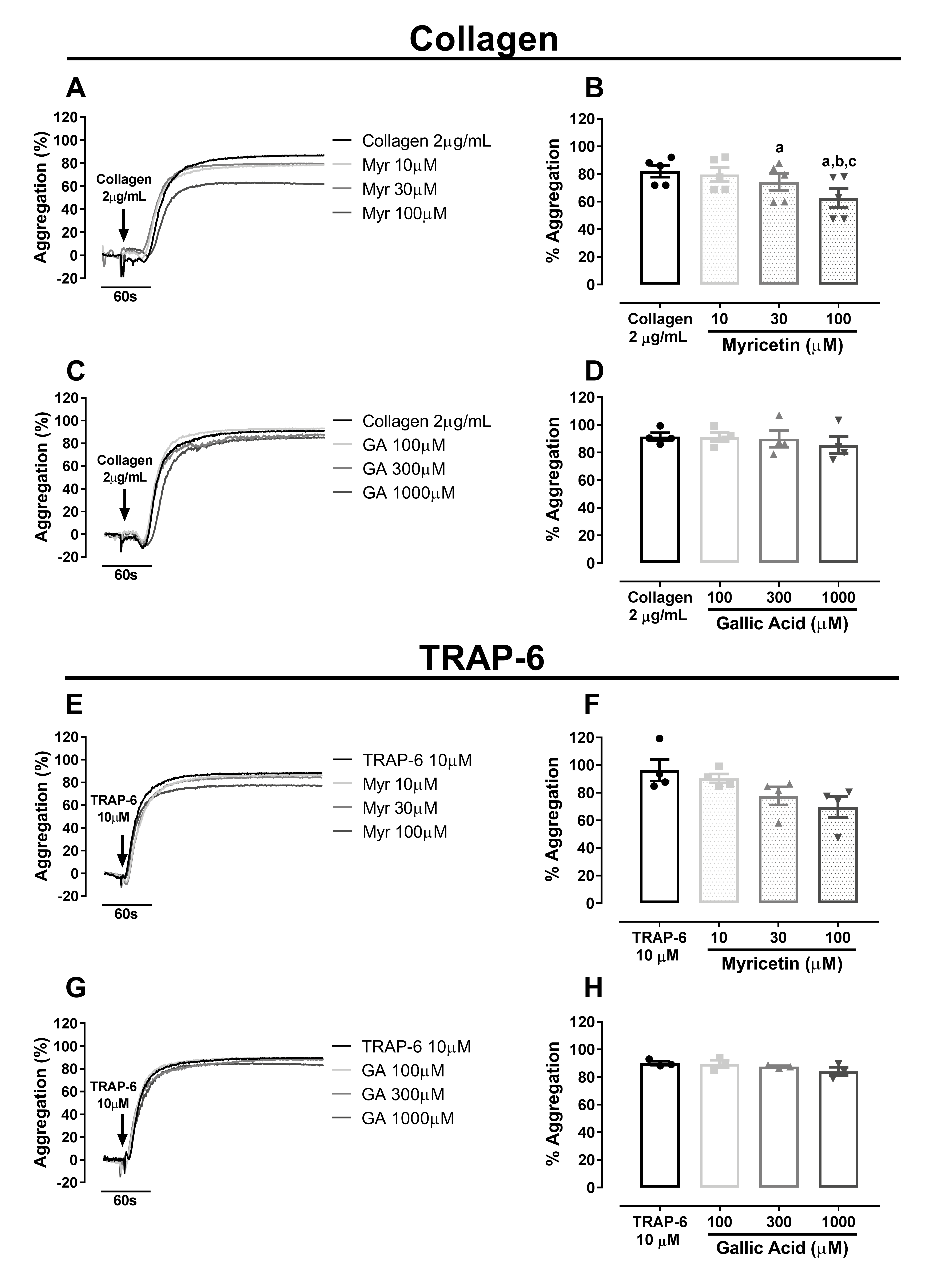

Supplement: Supplementary Figure 3 — Decreased effect of Myricetin in platelet-rich plasma. Platelet-rich plasma (PRP) was pre-treated with myricetin (Myr) or gallic acid (GA) for 10 minutes and stimulated with collagen or TRAP-6. (A) PRP treated with Myr and stimulated with collagen. (C) PRP treated with GA and stimulated with collagen. (E) PRP treated with Myr and stimulated with TRAP-6. (G) PRP treated with GA and stimulated with TRAP-6. Quantified data is shown right next to representative curves. a p<0.05 vs first column of graph. b p<0.05 vs second column of graph. c p<0.05 vs third column of graph. Data analysed by paired one-way ANOVA and Tukey as post-test. All bar graphs represent mean ± SEM and individual data points of at least 3 independent experiments. Arrows indicate when agonists were added. [file Image_3.tif]

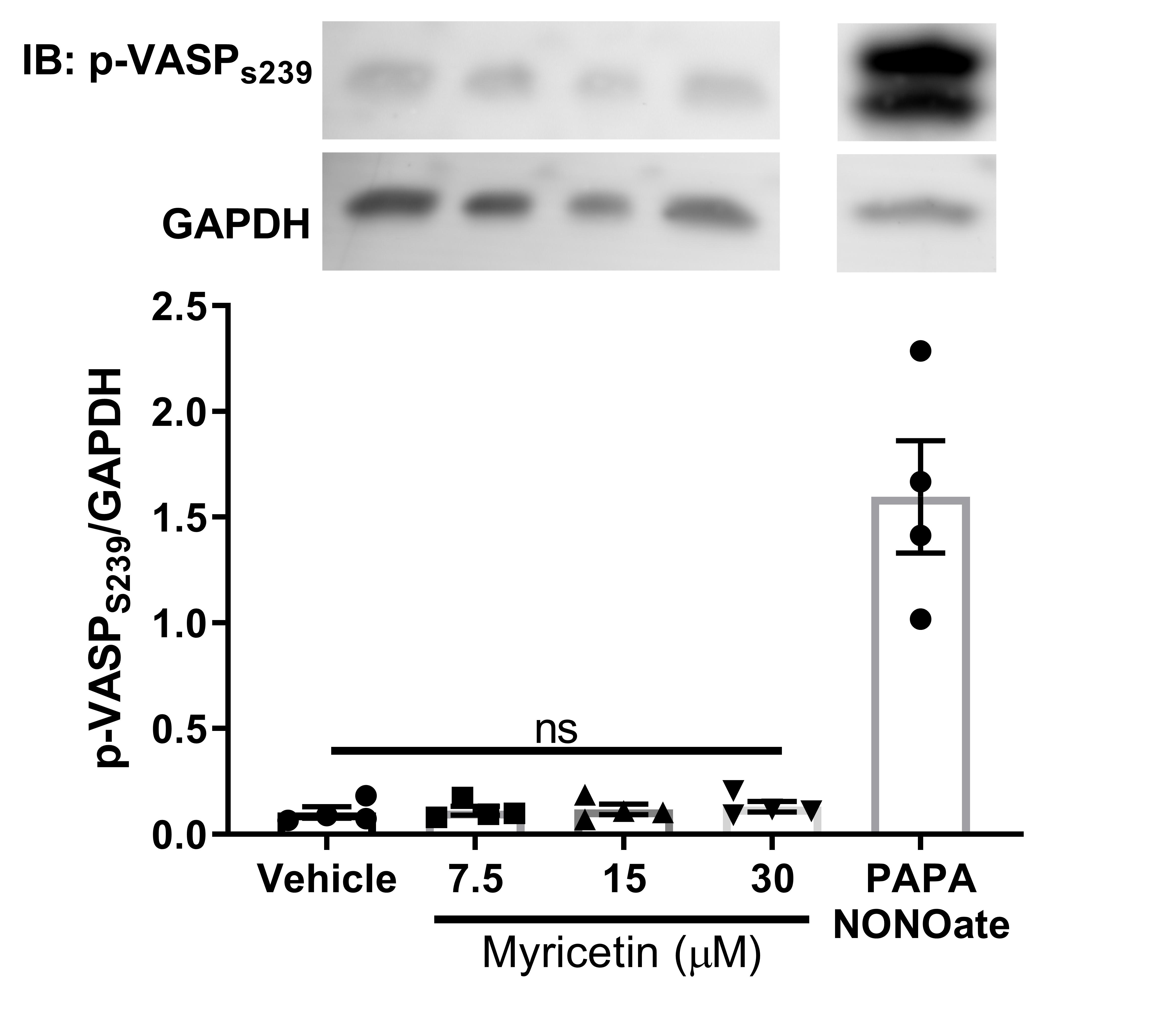

Supplement: Supplementary Figure 4 — Myricetin does not induce VASP phosphorylation. Resting WP were incubated with myricetin (7.5, 15 and 30 μM) or PAPA-NONOate (100 μM, positive control) for 10 minutes and lysed in laemmli buffer supplemented with reducing agent. Lysed cells were processed as described in Material and Methods and probed for VASPs239 and GAPDH as loading control. Bar graph represent present the mean of four independent experiments run and error bars indicate SEM. Data compared using One-way ANOVA followed by Tukey post-test. There were no statistical differences between groups. [file Image_4.jpeg]

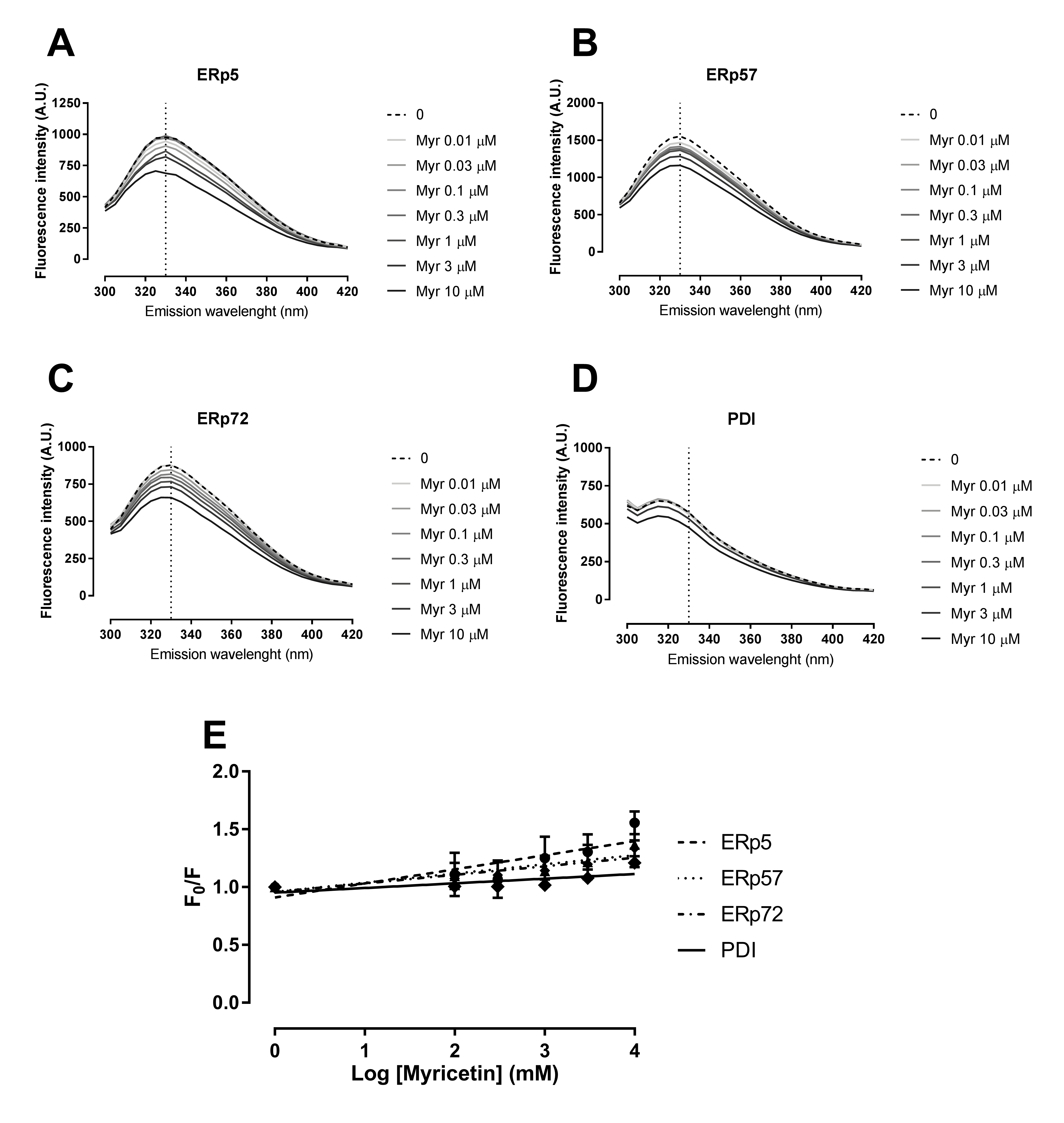

Supplement: Supplementary Figure 5 — Myricetin quenches fluorescence of ERp5, ERp57, ERp72 and PDI. Recombinant proteins were incubated with myricetin (0.01 to 10 μM) in a black 96-wells plate for 10 minutes and fluorescence spectra acquired in a fluorimeter using excitation set at 280 nm. Representative fluorescence spectra shown for ERp5 (A), ERp57 (B), ERp72 (C) and PDI (D). (E) Stern-volmer plot of quenching data is shown as the linear regression between F0/F and log of myricetin concentration in mM where F0 is the fluorescence of vehicle and F is the fluorescence in the presence of increasing concentrations of myricetin. Data represent at least three independent experiments run at least in duplicate and error bars indicate SEM. [file Image_5.tif]
